# Blood–Brain Barrier Permeability in Cases of Post-operative Delirium Is Associated with Central Nervous System Phosphatidylcholine Imbalances

**DOI:** 10.1007/s12035-026-05847-3

**Published:** 2026-04-21

**Authors:** Mijin Jung, Xiaobei Pan, Aoife Sweeney, Anthony P. Passmore, Bernadette McGuinness, Daniel F. McAuley, David Beverland, Amanda J. Heslegrave, Jonathan Schott, Henrik Zetterberg, Emma L. Cunningham, Brian D. Green

**Affiliations:** 1 School of Biological Sciences, Institute for Global Food Security, Queen’s University Belfast, 8 Malone Road, Belfast, Northern Ireland BT9 5BN UK; 2 Centre for Public Health, Institute of Clinical Sciences, Queen’s University Belfast, Block B, Royal Victoria Hospital Site, Grosvenor Road, Belfast, Northern Ireland BT12 6BA UK; 3 Centre for Experimental Medicine, Wellcome-Wolfson Institute for Experimental Medicine, Queen’s University Belfast, 97 Lisburn Road, Belfast, Northern Ireland BT9 7BL UK; 4 Outcomes Assessment Unit, Musgrave Park Hospital, Belfast Trust, Stockman’s Lane, Belfast, Northern Ireland BT9 7JB UK; 5 Department of Neurodegenerative Disease, UCL Institute of Neurology, Queen Square, London, WC1E 6BT UK; 6 UK Dementia Research Institute at UCL, London, WC1E 6BT UK; 7 Clinical Neurochemistry Laboratory, Sahlgrenska University Hospital, House V, Mölndal, S-431 80 Sweden; 8 Department of Psychiatry and Neurochemistry, Institute of Neuroscience and Physiology, The Sahlgrenska Academy at the University of Gothenburg, House V, Mölndal, S-431 80 Sweden; 9 Hong Kong Center for Neurodegenerative Diseases, Clear Water Bay, Hong Kong, China

**Keywords:** Blood-brain barrier (BBB), CSF/serum albumin ratio (Qalb), Phosphatidylcholine (PC), Delirium

## Abstract

Blood-brain barrier (BBB) permeability is reportedly increased in patients exhibiting postoperative delirium. Changes in either cerebrospinal fluid (CSF) or blood metabolites have also been reported in cases of delirium. This investigation examined whether BBB permeability influences metabolite concentrations on either side of the barrier, and whether this changes in cases of delirium. This study is a nested case-control cohort (*n* = 54) drawn from the entire cohort of *n* = 315 participants. Preoperative CSF and plasma from postoperative delirium and control participants matched for age and gender underwent metabolomic profiling. Concentrations of metabolites in plasma and in CSF were obtained, and where measurable, a CSF/plasma metabolite ratio (Qmetab) was calculated. CSF/plasma albumin ratio (Qalb), a recognized indicator of BBB permeability, was also calculated. The above parameters were compared between delirium and control groups. Finally, each Qmetab was correlated against Qalb for control or delirium cases. In cases where delirium would later occur there were significant elevations in the Qmetabs of amino acids (Gln, His, Met, Phe, Tyr and Val), and one phosphatidylcholine (PC). Many other PC Qmetabs significantly correlated with Qalb and this mainly occurred in individuals subsequently experiencing delirium. In persons prone to postoperative delirium, the equilibrium of certain species of PC across the BBB correlates with BBB permeability. It is unclear whether this occurs because of changes in PC metabolism or transit, but understanding this could improve our understanding of the mechanisms underlying delirium.

**Supplementary Information:**

The online version contains supplementary material available at 10.1007/s12035-026-05847-3.

## Introduction

Delirium is an acute clinical syndrome characterized by disturbances in attention, awareness, and cognition [1], which is common in older people following surgery [2, 3]. Delirium is associated with increased mortality and morbidity, particularly cognitive impairment [4, 5]. While several risk factors for delirium have been identified [6], the pathophysiological processes underlying delirium remain poorly understood. The identification of biomarkers for delirium is important to inform this inadequate pathophysiological understanding of delirium. Metabolomic analysis affords an opportunity to study the processes and pathways underlying delirium and combined with the advent of metabolomic evaluation of dementia-causing diseases, how delirium processes relate to neurodegeneration [7–9].

The association between vascular integrity and cognitive impairments has been studied as neuroinflammation, neurotoxicity, and imbalance in the brain environment [10, 11]. Vascular dysfunction is a risk factor for and has been implicated in the development and sequelae of delirium and dementia [12–14]. Several vascular risk factors, such as stroke, hypertension, and diabetes, and also genetic risk factors, such as *APOE*4, can be associated with defects in the vascular system leading to blood-brain barrier (BBB) impairment, which can also lead to cognitive decline [15–18]. The BBB is a highly selective physiological interface between the peripheral circulation and the central nervous system (CNS). It plays a critical role in maintaining brain homeostasis by tightly regulating the movement of solutes, metabolites, and immune cells. Previous studies demonstrated the relationship between BBB dysfunction and delirium from an inflammatory perspective, including levels of plasminogen activation inhibitor-1 (PAI-1), E-selection, angiopoietin-2 (Ang-2), S100B, and phosphorylated neurofilament heavy subunit (pNF-H) from serum in delirium patients [19–21].

Postoperative delirium (POD) commonly affects older adults following surgery and anesthesia. Study of elective surgical populations permits clinical, including cognitive and biomarker, characterization at baseline, when participants are well, and structured assessment of delirium symptoms within a predictable and relatively short postoperative window. Studies of postoperative delirium in elective surgical populations therefore facilitate the investigation of both the pathophysiology of delirium and how the incidence and sequelae of delirium sit in the wider neurodegeneration context.

Hence, a more detailed understanding of the impact of the permeability of BBB on the incidence of delirium is integral to our understanding of why delirium develops, especially after surgery and anesthesia, where drugs designed to cross the BBB are utilized. As the BBB acts as an essential physiological barrier separating the CNS from solutes in peripheral circulation, maintaining homeostasis in the brain environment, BBB permeability could increase brain vulnerability to delirium [22–24].

The association between BBB permeability and delirium has thus far centered on inflammation, such as disturbed cytokines or neural cell activities [19, 20, 25], but has rarely been studied from the perspective of the metabolome [23, 24]. Metabolites can serve as objective indicators of early pathological changes, and therefore metabolomic analyses could improve our understanding of the processes underpinning delirium [26, 27]. Watne et al. demonstrated higher levels of kynurenine pathway metabolites in the serum, and to a greater extent, CSF of trauma and medical participants with concurrent active delirium compared to trauma and control participants without delirium [28]. This provides evidence of levels during, but not present before, episodes of delirium. Tripp et al. compared the metabolomics profile of delirium and no delirium groups in an elective surgical cohort both preoperatively and at postoperative day 2, demonstrating differences in valine, leucine, isoleucine, and citrate metabolites, respectively, but this was in plasma only [29]. In a more recent study, Tripp et al. expanded their investigation to CSF using a multi-omics approach. They reported 26 lipids that differed in concentration between control and postoperative delirium [30] 

Given the complex relationship between BBB and lipids and the crucial role of lipid composition, particularly phospholipids, in maintaining BBB integrity and function, alterations in lipid metabolism may lead to BBB dysfunction and contribute to the development of neurological diseases [31, 32]. Phospholipids are key neuronal and glial membrane components, and their dysregulation has been associated with neuroinflammation, oxidative stress, and disrupted synaptic function [33, 34]. In studies of delirium, altered concentrations of several lipid species have been reported in both CSF and plasma samples, suggesting a potential role in delirium pathophysiology [30, 35–37].

The novel approach of the present investigation was to utilize matched CSF and plasma samples to measure and ratio the levels of metabolites on either side of the BBB. Then, it examined whether any differed in delirium-prone individuals, and also whether any were strongly related to BBB permeability (as measured by CSF/plasma albumin ratio (Qalb)). The findings clearly show that there are imbalances in a number of metabolites prior to the manifestation of delirium, and it could be postulated that these are linked to the increased permeability of the BBB.

## Materials and Methods

### Participants

Participants were sampled from an observational cohort study of *n* = 315 participants [38]. In brief, between March 2012 and October 2014, participants aged over 65 years and without a diagnosis of dementia scheduled for primary elective hip or knee arthroplasty were recruited. Written informed consent was obtained from all participants, and the study was approved by local ethical committee procedures (Office for Research Ethics Committees Northern Ireland; REC reference: 10/NIR01/5). Participants had CSF and plasma sampled preoperatively at the time of anesthetic administration. Participants were assessed daily postoperatively for delirium, using the Confusion Assessment Method and supplemented by notes review, as previously described [38–40].

Participants with sufficient matched CSF and plasma for analyses were eligible for this substudy. Included participants were matched for age and sex and divided into delirium (*n* = 28, aged 76.2 ± 5.7) and control (*n* = 26, aged 75.8 ± 5.2) groups [40]. The groups originally had *n* = 27 in each, but one participant was subsequently recategorized when further medical notes became available and were reviewed.

### Sample Collection and Preparation

Venous blood was sampled from fasted participants into lithium heparin bottles preoperatively at the time of anesthetic administration. All samples in an insulated icebox were transported to the laboratory on wet ice within 12 h of collection, where they were centrifuged at 4 °C at 3750 rpm for 10 min and 500 mL aliquots of the supernatant pipetted (Sarstedt, Germany, Catalog No. 70.762) into 1.5-mL polypropylene tubes (Sarstedt, Germany, Catalog No. 72.69.001) and stored at −80 °C.

Spinal anesthesia was carried out, fasting, in the sitting or lateral positions using a 25-gauge needle (diameter 0.53 mm; length 90 mm). Whitacre-type spinal needles with graduated metal introducers (Vygon). Once CSF was obtained, a 5-mL syringe (BD Plastipak; BD) was attached to the spinal needle and up to 5 mL of CSF was withdrawn. The CSF was immediately transferred to a 30=mL sterile polypropylene universal container (Unisurge) and placed in an insulated container on wet ice. All samples in an insulated icebox were transported to the laboratory within 12 h of collection. CSF samples were centrifuged at 4 °C at 3000 rpm/1811 g for 5 min with no brake and 500 µL aliquots of the supernatant pipetted (Sarstedt, Germany, Catalogue No. 70.762) into 1.5 mL polypropylene tubes (Sarstedt, Germany, Catalogue No. 72.69.001) and stored at −80 °C.

### Albumin Analysis

Matched CSF and preoperative plasma samples were analyzed for albumin concentration to calculate the CSF: plasma albumin ratio (Qalb). Samples were transported to the Kelvin Laboratories on dry ice in the Royal Victoria Hospital site, Belfast. There they were thawed at room temperature on a roller mixer. Samples were not centrifuged prior to analysis. The samples were analyzed on a Roche/Hitachi Cobas 8000 system with CSF and plasma samples run using the ALBC2:CAN 8412 and ALBS2:CAN 8128 reagent kits. Analyses were as per the manufacturer’s instructions. In brief, this immunoturbidimetric assay involves binding anti-human albumin antibodies in the reagent with albumin in the sample forming antigen/antibody complexes. The complexes are detected using turbidimetry, with the degree of turbidity being proportional to the concentration of albumin in the sample. CSF albumin concentration (g/L calculated from mg/L) was divided by plasma albumin concentration (g/L) to give the Qalb value, which was then expressed as Qalb × 10^3^.

### DNA Processing and Analysis

DNA was collected and processed to facilitate the analysis of Apolipoprotein E (*APOE*) genotype. Venous blood was collected into PAXgene Blood DNA tubes (Qiagen, catalogue number: 761125). DNA sample collection was conducted according to the manufacturer’s instructions (TaqMan Single Nucleotide Polymorphism Genotyping Assay; Life Technologies; Catalogue No. 4351379) and analysed using the TaqMan Single Nucleotide Polymorphism Genotyping Assay (Life Technologies; Catalogue No. 4351379) as per the manufacturer’s instructions. *APOE* status was inferred from the genotype at the two alleles, rs7412 and rs429358.

### Targeted Metabolomics

Quantitative mass spectrometry-based metabolomic profiling was carried out by the Biocrates Absolute IDQ p180 (BIOCRATES, Life Science AG, Innsbruck, Austria). All serum and CSF samples were prepared according to the manufacturer’s instructions and analyzed on a triple-quadrupole mass spectrometer (Xevo TQ-MS, Waters Corporation, Milford, USA). Metabolites (amino acids and biogenic amines) were derivatized using phenylisothiocyanate (PITC) in the presence of isotopically labelled internal standards, separated using a UPLC (I-Class, Waters Corporation, UK) system with a reverse-phase column (Waters ACQUITY UPLC BEH C18 2.1 × 50 mm, 1.7 µm; UK) and quantified using a triple-quadrupole mass spectrometer (Xevo TQ-MS, Waters Corporation, UK) operating in the multiple reaction monitoring (MRM) mode. All the remaining metabolites (acylcarnitines, hexoses, glycerophospholipids, and sphingolipids) were quantified using the same mass spectrometer without column separation by the flow injection analysis (FIA) operating in MRM mode. Metabolite concentrations were calculated and expressed as micromole (µM). The mean of the coefficient of variation (CV) for the 184 metabolites in repeated quality controls was 0.12, and 85% of the metabolites had a CV of < 0.15. Following these analyses, 59 metabolites were selected above LOD (limit of detection) values.

### Statistical Analysis

For statistical analysis, SPSS (version 26) was used for the normality test, Student’s *t*-test for parametric data, Mann–Whitney *U* test for non-parametric data to compare two given samples, and χ2 analysis and false discovery rates (FDR, q-value) were calculated based on Benjamini-Hochberg.

To determine the correlation between metabolite ratio and concentrations and Qalb, correlation coefficient r, *p*-value, and *q*-value were calculated. When it comes to the correlation model between metabolites (Qmetab, plasma metab, and CSF metab) and Qalb, *q*-values (*q* < 0.05) were focused more rather than *p*-values to discover more appropriate and reliable metabolites among 59 metabolites as *q*-value provides more restricted and controlled measure of the false positive discovery rate. The correlation coefficient *r* values and *p*-values were from either Pearson r correlation analysis for parametric values or Spearman r correlation analysis for non-parametric values. Prism 9 was used for correlation curves.

## Results

### Clinical Characteristics

The baseline characteristics of the delirium and control groups are shown in Table 1. Participants in the delirium group had significantly lower estimated IQ and Mini-Mental State Examination (MMSE) scores compared with controls (*p* = 0.041 and *p* = 0.012, respectively). Of note, while the difference in Qalb between the two groups is not statistically significant in the nested case–control cohort (*n* = 54), median Qalb was 6.13 (4.93–8.36) in the delirium group compared with 5.59 (4.41–6.59) in controls; it shows a significant value in the larger cohort with sufficient sample available (*n* = 244, Supplementary Table 1). It is of a similar magnitude to the difference between the delirium and control groups in the entire cohort, i.e., delirium (*n* = 35) 6.7 ± 3.0 versus controls (*n* = 209) 5.7 ± 2.5 (Mean ± SD, *p*-value = 0.03) [41].

**Table 1:** Baseline characteristics of control and delirium groups

	Control (*n *= 26)	Delirium (*n* = 28)	Statistical test	***p***-value	% Difference	AUC
Age, mean (SD)	75.8 (5.2)	76.2 (5.7)	T = 0.224	0.824	-	-
Sex, female (%)	14/26	15/28	X2 = 0	1.00	-	-
Apolipoprotein, *APOE*4 Yes (%)	8/26	9/27	X2 = 0	1.00	-	-
Charlson Comorbidity Index (CCI) 0/1/2/3	15/8/3/0	11/11/4/1	MWU = 286.5	0.209	-	-
Estimated IQ, mean (SD)	111.7 (7.5)	106.8 (9.3)	T = 2.093	0.041^*^	4.44%	0.643
Mini-Mental State Examination (MMSE) score, median (IQR)	28.0 (27.0–29.0)	26.0 (25.0–28.0)	MWU = 203	0.012^*^	6.26%	0.699
Albumin ratio (CSF/plasma), median (IQR)	5.59 (4.41–6.59)	6.13 (4.93–8.36)	MWU = 284	0.328	13.82%	0.580

Significant *p*-values are shown in bold. *p < 0.05 control vs delirium. *AUC* the area under the curve, *SD* standard deviation, *IQR* interquartile range, *T* Student’s *t*-test, *X2* chi-square test, *MWU* Mann–Whitney *U* test

### Comparison of CSF and Plasma Metabolite Concentrations Between Delirium and Control Groups

The concentrations of most metabolites in CSF are higher in the delirium group compared to the control group (Supplementary Table 2). Nine metabolites (glutamine, histamine, methionine, ornithine, serine, threonine, tyrosine, putrescine, and spermidine) showed a significance between control and delirium (*p* < 0.05), but with *q*-value added for false discovery rate, three metabolites glutamine, putrescine, and spermidine were significantly higher in the delirium group (*p *< 0.05, *q* < 0.05). Plasma metabolite concentrations differ in both directions, with lipids tending to be lower in delirium compared to control groups (Supplementary Table 3).

### Comparison of Qmetab Between Delirium and Control Groups

**Fig. 1:**
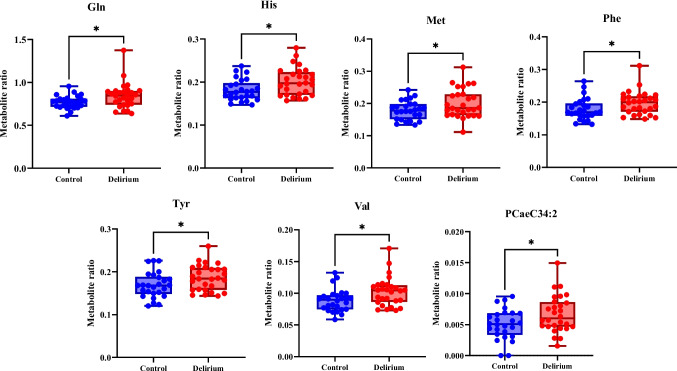
Metabolites metabolite ratios (Qmetabs) differing between control and delirium groups. *Gln* glutamine, *His* histidine, *Met* methionine, *Phe* phenylalanine, *Tyr* tyrosine, *Val* valine. Significant *p*-values are shown *p < 0.05 control vs delirium

Supplementary Table 4 shows Qmetab for delirium and control groups, the *p*/*q*-values, directions, and the % difference. Of the 59 metabolite ratios where both plasma and CSF concentrations were above the LOD, only 7 had a *p*-value less than 0.05. These were glutamine, histidine, methionine, phenylalanine, tyrosine, valine, and PCaeC34:2, all of which tended to be higher in cases of delirium (9.09–28.28%) (Fig. 1). However, none of the ratios was significant after multiple comparisons correction (*q* < 0.05).

### Correlation Between Qmetab and Qalb

**Fig. 2:**
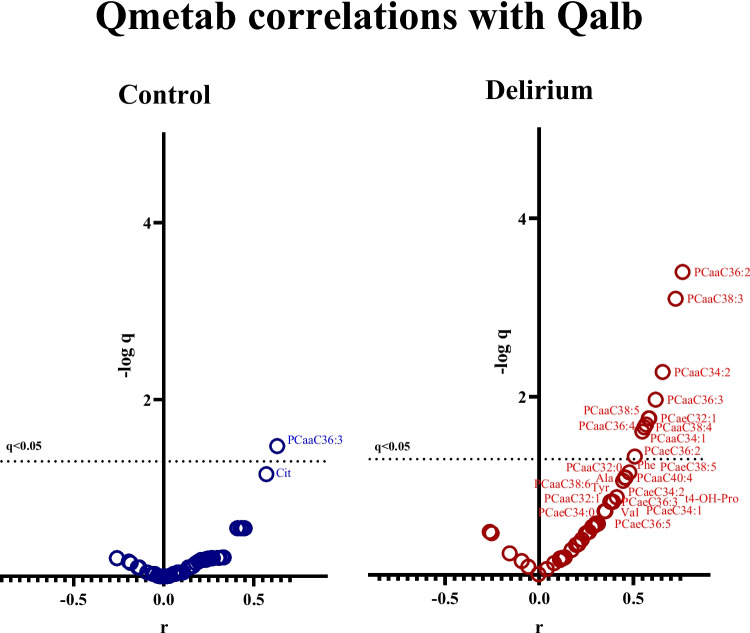
Correlation of Qmetab with Qalb. The panels show the correlations between each Qmetab and Qalb in control subjects (left) and in subjects later experiencing delirium. X-axes are correlation coefficients (r) from Spearman r, and the Y-axes are –log q. The dotted line shows the cutoff line (0.05) for the corrected *p*-value (q). Correlations above this line were deemed statistically significant

Results of the correlation analyses between all 59 available Qmetab and Qalb are shown in Supplementary Table 5. Figure 2 illustrates the extent to which Qmetab correlated with Qalb for control and delirium groups, with those above the dotted line being statistically significant (*q* < 0.05). Table 2 shows the 10 Qmetab that significantly correlated with Qalb (*r* > 0.4, *p* < 0.05, *q* < 0.05) across all participants. In addition, 16 Qmetab were correlated with Qalb in the delirium group (*r* > 0.4, *p* < 0.05), and 7 Qmetab were (*r* > 0.4, *p* < 0.05) in the control group (Supplementary Table 5). The ratio for PCaaC36:3 was the only one to correlate with Qalb in both delirium and control groups significantly. Table 2 contains all the metabolites indicated above the dotted line in control or delirium (*p* < 0.05, *q* < 0.05). Among metabolites in Table 2, we chose 10 phospholipids showing significant values (*p* < 0.05, *q* < 0.05) in the delirium group and investigated the comparison of phospholipids concentrations between the control and delirium groups; each of their metabolite concentration is displayed in Table 3. There was no significant difference between control and delirium in each plasma and CSF, but those phospholipids slightly decreased in delirium at the plasma level, whilst increasing in delirium at the CSF level.

**Table 2:** The correlation coefficient with Qalb with *p*-value, and *q*-value from the selected Qmetab

Metabolite	All participants			Control			Delirium		
	*r*	*p*-value	*q*-value	*r*	*p*-value	*q*-value	r	*p*-value	*q*-value
Ala	0.4496	0.0008^***^	0.0076^**^	0.4468	0.0221^*^	0.2858	0.4564	0.0191^*^	0.0810
PC aa C34:1	0.2601	0.0626	0.1273	−0.0229	0.9116	0.9960	0.5480	0.0038^**^	0.0246^*^
PC aa C34:2	0.5621	1.45E-05^***^	0.0003^***^	0.4277	0.0293	0.2858	0.6568	0.0003^***^	0.0053^**^
PC aa C36:2	0.5137	9.83E-05^***^	0.0015^**^	0.2171	0.2867	0.6499	0.7621	6.08E-06^***^	0.0004^***^
PC aa C36:3	0.6331	4.74E-07^***^	2.80E-05^***^	0.6294	0.0006^***^	0.0336^*^	0.6198	0.0007^***^	0.0108^*^
PC aa C36:4	0.4613	0.0006^***^	0.0068^**^	0.2732	0.1770	0.6226	0.5685	2.44E-03^***^	0.0206^*^
PC aa C38:3	0.5682	1.11E-05^***^	0.0003^***^	0.3265	0.1036	0.6109	0.7251	2.78E-05^***^	0.0008^***^
PC aa C38:4	0.4125	0.0024^**^	0.0141^*^	0.1460	0.4767	0.7896	0.5591	0.0030^**^	0.0221^*^
PC aa C38:5	0.4466	0.0009^***^	0.0076^**^	0.2465	0.2248	0.6315	0.5829	0.0018^**^	0.0175^*^
PC aa C38:6	0.4122	0.0024^**^	0.0141^*^	0.4117	0.0367^*^	0.2858	0.3812	0.0547	0.1513
PC ae C32:1	0.4376	0.0012^**^	0.0087^**^	0.2617	0.1965	0.6226	0.5857	0.0017^**^	0.0175^*^
PC ae C36:2	0.3359	0.0149^*^	0.0550	0.1583	0.4399	0.7633	0.5085	0.0080^**^	0.0471^*^

*r*-values are from Spearman correlation and represent the correlation coefficient r Qalb vs Qmetab. Significant *p*-values are shown in bold. *p < 0.05, **p < 0.01, ***p < 0.001 Qalb vs Qmetab. *q*-values are from Benjamini–Hochberg. Significant *q*-values are shown in bold. *q < 0.05, **q < 0.01, ***q < 0.001 Qalb vs Qmetab

**Table 3:** Comparison of metabolite concentration of glycerophospholipids between control and delirium in both plasma and CSF

Metabolite	Control (plasma)	Delirium (plasma)	↑↓	% difference	Control (CSF)	Delirium (CSF)	↑↓	% difference
	Mean ± SD (g/L)	Mean ± SD (g/L)			Mean ± SD (mg/L)	Mean ± SD (mg/L)		
PC aa C34:1	224.1 ± 56.52	217.5 ± 59.13	↓	2.98	1.924 ± 0.471	2.003 ± 0.567	↑	4.04
PC aa C34:2	332.2 ± 99.46	296.1 ± 60.02	↓	11.49	0.180 ± 0.051	0.210 ± 0.147	↑	15.16
PC aa C36:2	179.0 ± 46.17	167.8 ± 29.81	↓	6.42	0.183 ± 0.065	0.213 ± 0.116	↑	15.11
PC aa C36:3	108.2 ± 29.04	101.2 ± 18.72	↓	6.68	0.067 ± 0.028	0.087 ± 0.062	↑	26.64
PC aa C36:4	171.0 ± 56.17	163.8 ± 42.82	↓	4.35	0.181 ± 0.058	0.226 ± 0.154	↑	22.10
PC aa C38:3	43.17 ± 12.70	39.98 ± 8.68	↓	7.66	0.049 ± 0.024	0.058 ± 0.028	↑	16.48
PC aa C38:4	90.27 ± 29.84	86.75 ± 24.43	↓	3.97	0.172 ± 0.052	0.201 ± 0.112	↑	15.25
PC aa C38:5	46.83 ± 13.20	45.29 ± 12.38	↓	3.33	0.048 ± 0.019	0.056 ± 0.031	↑	15.30
PC ae C32:1	2.333 ± 0.641	2.344 ± 0.550	≈	0.50	0.018 ± 0.009	0.020 ± 0.013	↑	14.16
PC ae C36:2	9.617 ± 2.613	9.844 ± 2.548	↑	2.33	0.019 ± 0.010	0.020 ± 0.013	↑	4.87

*CSF*, cerebrospinal fluid; *SD*, standard deviation

### APOE ε4 Carrier and CSF Lipids

A comparison of concentrations of lipids in CSF was carried out according to whether they were *APOE* ε4 allele carriers (Yes/No), which are related to lipid metabolism. Only CSF lipids were considered to identify if lipid alterations correlated with APOE ε4 carrier. As shown, none of them was statistically significant (Supplementary Table 6).

## Discussion

The BBB is the key interface between the peripheral blood circulation and the central nervous system (CNS), controlling the access of circulating molecules from the blood to the CNS [42]. The permeability of this barrier increases with age, thus decreasing protection of the CNS, potentially causing metabolic disequilibrium in the brain, or exposing it to various toxins. Increased BBB permeability could be the result of, and/or a contributing factor to, the development of neurodegenerative diseases [43, 44].

Delirium is a common postoperative complication and is associated with both pre-existing and subsequent neurodegeneration and cognitive impairment. The statistically significant differences in estimated IQ and MMSE between the control and delirium groups, even in this small study, reflect these well-established associations [38, 39]. Interpretation of our results should therefore consider that delirium and control groups likely differ at baseline in education comorbidities, cognitive function, and neurodegeneration. Improving our pathophysiological understanding of delirium could lead to the identification of biomarkers and/or prevention strategies for both delirium and neurodegeneration. In this study, Qalb (the CSF/plasma albumin ratio) was used to assess BBB permeability in a nested case–control postoperative delirium cohort. Although Qalb significantly differed between control and delirium across the parent cohort (control: *n* = 209, mean 5.7; delirium: *n* = 35, mean 6.7; *p* = 0.03), this was not the case in the nested case–control cohort (control: *n* = 26, mean 5.6; delirium *n* = 28, mean 6.1; *p* = 0.33).

The concept that BBB dysfunction occurs in delirium is no longer controversial. A number of geographically distinct delirium cohorts have reported this to be an important pre- and postoperative factor [45]. Previous studies have elucidated the relationship between BBB dysfunction and delirium from an inflammation perspective [19–21, 46]. There are very few investigations of how increased BBB permeability affects metabolites, although one study focusing on diagnosed cases of AD reported that CSF levels of amino acids and acylcarnitines were positively correlated with Qalb [47]. Conversely, some metabolites, such as kynurenic acid and creatinine, were negatively correlated with Qalb in these AD patients [21]. This study also noted that AD patients had higher blood levels of citric acid cycle intermediates and long-chain acylcarnitines, but lower levels of amino acids [47]. These alterations appear to be of systemic origin and are mirrored in the CNS as a function of BBB permeability.

The present study capitalized on a relatively unique resource available at the Belfast postoperative delirium cohort - i.e., preoperatively collected, matched CSF and plasma samples. This meant that metabolomic profiling could be performed in either side of the BBB; the measured metabolites could then be ratioed to obtain a “Qmetab,” effectively a small molecule equivalent of the Qalb indicator used to assess BBB permeability. From here, it was then possible to assess which Qmetabs differed in delirium-prone individuals, and rather interestingly, whether any closely correlated with Qalb. The findings indicate that there are imbalances in a number of metabolites prior to the manifestation of delirium and that a number of other metabolites (chiefly PCs) are linked to the increased permeability of the BBB in delirium.

Of the 59 CSF/plasma metabolite ratios calculated, just seven (glutamine, histidine, methionine, phenylalanine, tyrosine, valine, and PCaeC34:2) were elevated in delirium at the *p* < 0.05 level, including aromatic amino acids that are precursors of monoamine neurotransmitters. Unfortunately, these did not withstand false discovery rate correction (*q* < 0.05), which may increase the risk of type II error for biologically related metabolites. There were 3 CSF metabolites (glutamine, putrescine, and spermidine) that were significantly higher in delirium (*q* < 0.05), a finding which our group has previously reported [40]. These will not be discussed further in the present article, but sufficed to say, these are potential biomarkers of delirium and implicate arginine metabolism in pathways predisposed to delirium [40].

However, the most striking finding of the present study is that the CSF/plasma ratio of several phospholipids strongly correlated to Qalb in delirium cases, and not in control cases. The BBB disruption of delirium cases related more to changes in CSF phospholipids than changes in plasma phospholipids. The BBB is a highly selective permeable cellular phospholipid protein bilayer barrier, and it has been theorized that BBB disruption in persons prone to delirium is an indicator of neurodegeneration [48]. Here, elevating CSF phospholipid and/or decreasing phospholipid levels could indicate the early stages of declining BBB function in persons prone to delirium. Further evaluation is needed to understand what phospholipid imbalances across the BBB mean for neurodegenerative processes, which are likely to increase delirium susceptibility, and/or pathophysiological processes leading to delirium. At present, these phospholipid imbalances do not appear to be related to the frequency of the *APOE* ε4 alleles.

It is well established that the brain is a lipid-rich organ containing high levels of these types of lipids [48, 49], which are important components of cell membranes. Several studies have suggested that alterations in phospholipids are related to neuronal injury, neuroinflammation, and neurodegeneration [50–53]. One study found that plasma phospholipid levels could predict memory impairment in AD, finding that ten plasma metabolites (PCaaC36:6, PCaaC38:0, PCaaC38:6, PCaaC40:1, PCaaC40:2, PCaaC40:6, PCaeC40:6, lysoPCaC18:2, C3, and C16:1-OH) were depleted in mild cognitive impairment (MCI) patients who converted to AD [54]. This finding is of interest given that plasma phospholipids in the present study were slightly lower (albeit not significantly) in delirium. However, these findings remain somewhat controversial, as larger cohort and meta-analysis studies have suggested that associations between phosphatidylcholine and AD risk are often modest and cohort-specific, highlighting challenges in reproducibility and the need for validation across diverse populations analytic platforms [55].

Regarding lipid alterations in delirium, recent studies have identified several lipid species associated with delirium. Han et al. reported 33 dysregulated lipid species in CSF, primarily related to glycerophospholipid metabolism and sphingolipid pathways. Among these, phosphatidylethanolamine (PE 40:7e) emerged as a strong predictive biomarker for POD patients (AUC = 0.92) [36]. Similarly, Huang et al. found that phosphatidylinositol species (PI [18:0/18:2], PI [20:4/18:0], and PI [18:1/20:3]) were significantly downregulated in POD patients [56]. Additionally, a recent Mendelian randomization study by He et al. suggested a potential causal role for lipid metabolites in delirium risk. This study identified a lipid ratio (phosphate to oleoyl-linoleoyl‑glycerol, 18:1/18:2) that was positively associated with delirium risk and highlighted phosphatidylinositol phosphate metabolism as a key pathway [37].

Beyond lipids, several metabolomics studies have reported alterations in amino acids (tryptophan, tyrosine, phenylalanine), acylcarnitine, and polyamines (glutamine, putrescine, and spermidine) in delirium. These findings suggest broader impairments in metabolic homeostasis, including dysregulated amino acid metabolism, oxidative stress, and mitochondrial dysfunction in delirium pathophysiology [40, 57–59]. While our focus is on phospholipid metabolism, we acknowledge the broader metabolomics studies and future studies integrate lipidomic findings with other metabolic domains to build a more comprehensive understanding of delirium pathology.

Given that the plasma and CSF samples here were collected preoperative (meaning that no patients had symptoms of delirium at the time of collection), it may be useful to understand whether phospholipid levels could significantly differ if delirium cases were followed up over a more protracted period of time. Furthermore, the mechanistic link between BBB permeability and phospholipid dysregulation in delirium is not yet fully understood. Our study addresses this gap by exploring preoperative phospholipid profiles in relation to subsequent delirium occurrence, contributing to building up the evidence suggesting that lipid imbalance may play a role in delirium pathophysiology.

The sample size of the nested case–control study is a potential limitation reducing the statistical power to detect differences between delirium and control groups. However, now that these metabolite ratios have been identified and their variances are now known, it will be possible to design larger, more sufficiently powered cohorts. Nonetheless, metabolite profiling across the BBB, particularly where there is dysfunction, represents an extremely novel approach to understanding the relationship between delirium and neurodegeneration.

In conclusion, BBB dysfunction is known to occur in delirium cases, and this study has identified changes in metabolite gradients across the barrier, and these could result from the alteration of BBB permeability. However, increased CSF metabolite concentrations may not exclusively reflect BBB leakage, as altered CSF water dynamics could also contribute to higher solute concentrations. In persons prone to postoperative delirium, the equilibrium of certain species of phosphatidylcholine across the BBB correlates with BBB permeability. Although it is unclear whether this occurs because of changes in PC metabolism or transit, an improved understanding of this could help unpick the processes and mechanisms underlying delirium.

## Supplementary Information

Below is the link to the electronic supplementary material.

ESM 1 (DOCX 15.8 KB)

ESM 2 (DOCX 24.1 KB)

ESM 3 (DOCX 24.0 KB)

ESM 4 (DOCX 23.2 KB)

ESM 5 (DOCX 26.6 KB)

ESM 6 (DOCX 16.4 KB)

## Data Availability

Data are available from the authors upon reasonable request.
