# Supplementary material for: Blood–Brain Barrier Permeability in Cases of Post-operative Delirium Is Associated with Central Nervous System Phosphatidylcholine Imbalances
Source: Mol Neurobiol. 2026 Apr 21;63(1):575. doi: 10.1007/s12035-026-05847-3 (PMC13099853; doi:10.1007/s12035-026-05847-3)
Supplement: Supplementary file 1 — (DOCX 15.8 KB) [file 12035_2026_5847_MOESM1_ESM.docx]

**Supplementary Table 1.** Baseline characteristics of control and delirium groups in the full cohort

|  | **Delirium (n = 35)** | **Control (n = 209)** | **statistics** | **p-value** | **% Difference** | **AUC (95% CI)** |
| --- | --- | --- | --- | --- | --- | --- |
| Age, mean (SD) | 76.3 (5.9) | 73.7 (5.7) | MWU= 2729 | 0.02 | - | - |
| Sex, female (%) | 19 /35 | 119 /209 | X2= 0.09 | 0.77 | - | - |
| Albumin ratio (CSF/plasma), median (IQR) | 6.75 (4.73 –8.39) | 5.72 (3.82 –7.12) | MWU=2784 | 0.02 | 20.50% | 0.619 (0.525 – 0.714) |

Significant p-values are shown in bold. *p < 0.05 control vs delirium. AUC: the area under the curve; CI: confidence interval; SD: standard deviation; IQR: interquartile range; T: Student’s t.test; X2: Chi-square test; MWU: Mann-Whitney U test.
