# Supplementary material for: Blood–Brain Barrier Permeability in Cases of Post-operative Delirium Is Associated with Central Nervous System Phosphatidylcholine Imbalances
Source: Mol Neurobiol. 2026 Apr 21;63(1):575. doi: 10.1007/s12035-026-05847-3 (PMC13099853; doi:10.1007/s12035-026-05847-3)
Supplement: Supplementary file 2 — (DOCX 24.1 KB) [file 12035_2026_5847_MOESM2_ESM.docx]

**Supplementary Table 2. Univariate analysis between control and delirium in concentration in CSF**

| Metabolites | Control | Delirium | p-value | q-value | ↑/↓ | % difference |
| --- | --- | --- | --- | --- | --- | --- |
|  | Mean (SD) | Mean (SD) |  |  |  |  |
| C3 | 0.026 (0.014) | 0.029 (0.018) | 0.363 | 0.663 | ↑ | 10.76% |
| Ala | 40.45 (10.49) | 41.45 (12.18) | 0.748 | 0.906 | ↑ | 2.45% |
| Arg | 23.12 (4.43) | 23.55 (4.71) | 0.731 | 0.906 | ↑ | 1.85% |
| Asn | 6.295 (1.118) | 6.612 (1.552) | 0.659 | 0.904 | ↑ | 4.91% |
| Asp | 1.291 (0.071) | 1.277 (0.087) | 0.354 | 0.663 | ↓ | 1.09% |
| Cit | 2.693 (0.756) | 2.880 (1.078) | 0.634 | 0.891 | ↑ | 6.72% |
| Gln | 549.6 (66.3) | 613.4 (66.6) | **2.11E-04^***^** | **0.012^*^** | ↑ | 10.97% |
| Glu | 1.145 (0.146) | 1.216 (0.199) | 0.105 | 0.344 | ↑ | 6.02% |
| Gly | 8.827 (1.820) | 10.67 (5.54) | 0.073 | 0.303 | ↑ | 18.86% |
| His | 13.12 (2.21) | 14.09 (1.73) | **0.013^*^** | 0.110 | ↑ | 7.19% |
| Ile | 6.343 (1.496) | 7.096 (2.233) | 0.332 | 0.663 | ↑ | 11.21% |
| Leu | 14.51 (3.81) | 16.01 (5.34) | 0.373 | 0.663 | ↑ | 9.82% |
| Lys | 28.18 (5.60) | 31.45 (6.39) | 0.077 | 0.303 | ↑ | 10.98% |
| Met | 3.694 (0.748) | 4.170 (0.877) | **0.037^*^** | 0.243 | ↑ | 12.11% |
| Orn | 4.175 (0.973) | 4.916 (1.010) | **0.008^**^** | 0.089 | ↑ | 16.31% |
| Phe | 10.59 (2.28) | 11.39 (2.47) | 0.100 | 0.344 | ↑ | 7.25% |
| Pro | 0.735 (0.325) | 0.919 (0.520) | 0.229 | 0.614 | ↑ | 22.26% |
| Ser | 23.88 (3.70) | 26.59 (3.60) | **0.009^**^** | 0.089 | ↑ | 10.73% |
| Thr | 25.47 (5.92) | 30.08 (6.49) | **0.009^**^** | 0.089 | ↑ | 16.57% |
| Trp | 2.507 (0.425) | 2.748 (0.443) | 0.043 | 0.254 | ↑ | 9.16% |
| Tyr | 9.003 (2.453) | 10.41 (2.65) | **0.016^*^** | 0.118 | ↑ | 14.46% |
| Val | 17.03 (3.28) | 20.15 (6.09) | 0.060 | 0.295 | ↑ | 16.76% |
| ADMA | 0.061 (0.024) | 0.067 (0.016) | 0.318 | 0.663 | ↑ | 9.04% |
| Creatinine | 96.45 (20.09) | 94.88 (23.44) | 0.580 | 0.856 | ↓ | 1.65% |
| Kynurenine | 0.170 (0.018) | 0.185 (0.033) | 0.088 | 0.325 | ↑ | 8.37% |
| Met-SO | 0.083 (0.031) | 0.089 (0.035) | 0.264 | 0.623 | ↑ | 6.87% |
| Putrescine | 0.115 (0.035) | 0.151 (0.044) | **0.001^*^** | **0.020^*^** | ↑ | 27.32% |
| Spermidine | 0.071 (0.012) | 0.085 (0.014) | **0.001^*^** | **0.020^*^** | ↑ | 18.97% |
| Spermine | 0.072 (0.002) | 0.073 (0.002) | 0.306 | 0.663 | ≈ | 0.63% |
| t4-OH-Pro | 0.573 (0.200) | 0.645 (0.180) | 0.153 | 0.475 | ↑ | 11.94% |
| Taurine | 7.265 (1.880) | 7.907 (1.652) | 0.188 | 0.555 | ↑ | 8.45% |
| SDMA | 0.200 (0.075) | 0.193 (0.064) | 0.768 | 0.906 | ↓ | 3.45% |
| PCaaC32:0 | 0.318 (0.087) | 0.314 (0.092) | 0.879 | 0.938 | ↓ | 1.17% |
| PCaaC32:1 | 0.134 (0.038) | 0.151 (0.049) | 0.250 | 0.623 | ↑ | 11.30% |
| PCaaC34:1 | 1.924 (0.471) | 2.003 (0.567) | 0.835 | 0.928 | ↑ | 4.04% |
| PCaaC34:2 | 0.180 (0.051) | 0.210 (0.147) | 0.710 | 0.906 | ↑ | 15.16% |
| PCaaC36:1 | 0.222 (0.055) | 0.233 (0.075) | 0.566 | 0.856 | ↑ | 4.60% |
| PCaaC36:2 | 0.183 (0.065) | 0.213 (0.116) | 0.436 | 0.715 | ↑ | 15.11% |
| PCaaC36:3 | 0.067 (0.028) | 0.087 (0.062) | 0.287 | 0.651 | ↑ | 26.64% |
| PCaaC36:4 | 0.181 (0.058) | 0.226 (0.154) | 0.354 | 0.663 | ↑ | 22.10% |
| PCaaC38:3 | 0.049 (0.024) | 0.058 (0.028) | 0.203 | 0.570 | ↑ | 16.48% |
| PCaaC38:4 | 0.172 (0.052) | 0.201 (0.112) | 0.494 | 0.788 | ↑ | 15.25% |
| PCaaC38:5 | 0.048 (0.019) | 0.056 (0.031) | 0.382 | 0.663 | ↑ | 15.30% |
| PCaaC38:6 | 0.047 (0.022) | 0.068 (0.062) | 0.076 | 0.303 | ↑ | 37.05% |
| PCaaC40:4 | 0.012 (0.009) | 0.014 (0.010) | 0.602 | 0.866 | ↑ | 14.25% |
| PCaaC40:5 | 0.016 (0.011) | 0.016 (0.012) | 0.788 | 0.912 | ≈ | 0.79% |
| PCaeC32:1 | 0.018 (0.009) | 0.020 (0.013) | 0.573 | 0.856 | ↑ | 13.23% |
| PCaeC34:0 | 0.014 (0.008) | 0.013 (0.009) | 0.838 | 0.928 | ↓ | 3.63% |
| PCaeC34:1 | 0.064 (0.015) | 0.068 (0.025) | 0.890 | 0.938 | ↑ | 6.05% |
| PCaeC34:2 | 0.033 (0.014) | 0.046 (0.026) | 0.060 | 0.295 | ↑ | 32.68% |
| PCaeC36:1 | 0.036 (0.017) | 0.043 (0.029) | 0.742 | 0.906 | ↑ | 17.24% |
| PCaeC36:2 | 0.019 (0.010) | 0.020 (0.013) | 0.755 | 0.906 | ↑ | 4.87% |
| PCaeC36:3 | 0.010 (0.006) | 0.012 (0.013) | 0.958 | 0.979 | ↑ | 19.29% |
| PCaeC36:5 | 0.016 (0.009) | 0.016 (0.012) | 0.979 | 0.979 | ↑ | 2.88% |
| PCaeC38:4 | 0.015 (0.009) | 0.017 (0.014) | 0.849 | 0.928 | ↑ | 11.88% |
| PCaeC38:5 | 0.016 (0.010) | 0.021 (0.012) | 0.263 | 0.623 | ↑ | 25.30% |
| SMC16:0 | 0.165 (0.056) | 0.162 (0.073) | 0.431 | 0.715 | ↓ | 2.28% |
| SMC18:0 | 0.166 (0.060) | 0.162 (0.051) | 0.979 | 0.979 | ↓ | 2.06% |
| H1 | 4050.7 (2101.3) | 4048.6 (1294.2) | 0.716 | 0.906 | ≈ | 0.05% |

Significant p-values are shown in bold. *p < 0.05, **p < 0.01, *** p < 0.001 control vs delirium. q-values are from Benjamini–Hochberg. Significant q-values are shown in bold. *q < 0.05 control vs delirium. SD: standard deviation; Ala: alanine; Arg: arginine; Asn: asparagine; Asp: aspartate; Cit: citrulline; Gln: glutamine; Glu: glutamate; Gly: glycine; His: histidine; Ile: isoleucine; Leu: leucine; Lys: lysine; Met: methionine; Orn: ornithine; Phe: phenylalanine; Pro: proline; Ser: serine; Thr: threonine; Trp: tryptophan; Tyr: tyrosine; Val: valine; ADMA: asymmetric dimethylarginine; SDMA: symmetric dimethylarginine; H1: hexose.
