# Supplementary material for: Blood–Brain Barrier Permeability in Cases of Post-operative Delirium Is Associated with Central Nervous System Phosphatidylcholine Imbalances
Source: Mol Neurobiol. 2026 Apr 21;63(1):575. doi: 10.1007/s12035-026-05847-3 (PMC13099853; doi:10.1007/s12035-026-05847-3)
Supplement: Supplementary file 3 — (DOCX 24.0 KB) [file 12035_2026_5847_MOESM3_ESM.docx]

**Supplementary Table 3. Univariate analysis between control and delirium in concentration in plasma**

| Metabolites | Control | Delirium | p-value | q-value | ↑/↓ | % difference |
| --- | --- | --- | --- | --- | --- | --- |
|  | Mean (SD) | Mean (SD) |  |  |  |  |
| C3 | 0.208 (0.080) | 0.241 (0.097) | 0.197 | 0.939 | ↑ | 14.85% |
| Ala | 346.3 (75.1) | 333.8 (88.97) | 0.580 | 0.939 | ↓ | 3.68% |
| Arg | 84.14 (15.79) | 80.87 (16.94) | 0.468 | 0.939 | ↓ | 3.96% |
| Asn | 37.65 (6.34) | 38.05 (6.91) | 0.827 | 0.941 | ↑ | 1.05% |
| Asp | 4.054 (2.996) | 3.853 (1.153) | 0.829 | 0.941 | ↓ | 5.10% |
| Cit | 33.10 (7.11) | 34.70 (8.64) | 0.464 | 0.939 | ↑ | 4.71% |
| Gln | 715.8 (67.58) | 738.9 (91.40) | 0.301 | 0.939 | ↑ | 3.16% |
| Glu | 42.04 (19.64) | 41.70 (17.35) | 0.829 | 0.941 | ≈ | 0.83% |
| Gly | 198.9 (57.76) | 235.0 (96.88) | 0.213 | 0.939 | ↑ | 16.66% |
| His | 72.48 (6.81) | 70.98 (9.39) | 0.504 | 0.939 | ↓ | 2.08% |
| Ile | 68.73 (14.73) | 67.45 (13.25) | 0.739 | 0.939 | ↓ | 1.87% |
| Leu | 138.1 (35.72) | 134.0 (33.00) | 0.662 | 0.939 | ↓ | 3.02% |
| Lys | 159.5 (23.23) | 174.1 (25.84) | **0.034^*^** | 0.787 | ↑ | 8.76% |
| Met | 20.71 (2.89) | 21.21 (3.78) | 0.590 | 0.939 | ↑ | 2.38% |
| Orn | 47.69 (12.24) | 56.10 (13.33) | **0.020^*^** | 0.787 | ↑ | 16.20% |
| Phe | 59.79 (8.65) | 58.30 (10.38) | 0.572 | 0.939 | ↓ | 2.51% |
| Pro | 177.7 (32.94) | 197.6 (35.97) | **0.040^*^** | 0.787 | ↑ | 10.57% |
| Ser | 83.83 (17.05) | 88.20 (20.43) | 0.399 | 0.939 | ↑ | 5.08% |
| Thr | 86.87 (19.11) | 98.60 (23.89) | 0.111 | 0.939 | ↑ | 12.65% |
| Trp | 46.29 (10.15) | 48.75 (10.24) | 0.381 | 0.939 | ↑ | 5.16% |
| Tyr | 53.97 (14.02) | 56.50 (12.82) | 0.416 | 0.939 | ↑ | 4.59% |
| Val | 194.3 (28.09) | 197.3 (39.93) | 0.748 | 0.939 | ↑ | 1.54% |
| ADMA | 0.444 (0.229) | 0.389 (0.178) | 0.311 | 0.939 | ↓ | 13.14% |
| Creatinine | 70.08 (29.65) | 63.15 (22.37) | 0.568 | 0.939 | ↓ | 10.41% |
| Kynurenine | 2.634 (0.687) | 2.821 (0.699) | 0.239 | 0.939 | ↑ | 6.85% |
| Met-SO | 0.987 (0.394) | 0.937 (0.408) | 0.775 | 0.941 | ↓ | 5.19% |
| Putrescine | 0.107 (0.040) | 0.113 (0.042) | 0.591 | 0.939 | ↑ | 5.62% |
| Spermidine | 0.162 (0.099) | 0.163 (0.086) | 0.203 | 0.939 | ≈ | 0.68% |
| Spermine | 0.183 (0.041) | 0.187 (0.025) | 0.076 | 0.897 | ↑ | 1.86% |
| t4-OH-Pro | 9.224 (3.056) | 10.27 (4.41) | 0.782 | 0.941 | ↑ | 10.76% |
| Taurine | 56.74 (14.01) | 54.79 (11.79) | 0.581 | 0.939 | ↓ | 3.50% |
| SDMA | 0.695 (0.492) | 0.557 (0.285) | 0.436 | 0.939 | ↓ | 21.96% |
| PCaaC32:0 | 10.20 (2.89) | 10.48 (2.81) | 0.714 | 0.939 | ↑ | 2.77% |
| PCaaC32:1 | 17.22 (7.42) | 15.89 (5.72) | 0.890 | 0.962 | ↓ | 8.02% |
| PCaaC34:1 | 224.1 (56.52) | 217.5 (59.13) | 0.678 | 0.939 | ↓ | 2.98% |
| PCaaC34:2 | 332.2 (99.46) | 296.1 (60.02) | 0.118 | 0.939 | ↓ | 11.49% |
| PCaaC36:1 | 38.59 (9.99) | 39.17 (12.24) | 0.850 | 0.946 | ↑ | 1.49% |
| PCaaC36:2 | 179.0 (46.17) | 167.8 (29.81) | 0.302 | 0.939 | ↓ | 6.42% |
| PCaaC36:3 | 108.2 (29.04) | 101.2 (18.72) | 0.303 | 0.939 | ↓ | 6.68% |
| PCaaC36:4 | 171.0 (56.17) | 163.8 (42.82) | 0.593 | 0.939 | ↓ | 4.35% |
| PCaaC38:3 | 43.17 (12.70) | 39.98 (8.68) | 0.284 | 0.939 | ↓ | 7.66% |
| PCaaC38:4 | 90.27 (29.84) | 86.75 (24.43) | 0.637 | 0.939 | ↓ | 3.97% |
| PCaaC38:5 | 46.83 (13.20) | 45.29 (12.38) | 0.661 | 0.939 | ↓ | 3.33% |
| PCaaC38:6 | 65.91 (18.28) | 61.46 (16.07) | 0.346 | 0.939 | ↓ | 6.98% |
| PCaaC40:4 | 2.652 (0.677) | 2.566 (0.710) | 0.653 | 0.939 | ↓ | 3.28% |
| PCaaC40:5 | 9.338 (2.706) | 8.649 (2.357) | 0.322 | 0.939 | ↓ | 7.66% |
| PCaeC32:1 | 2.333 (0.641) | 2.344 (0.550) | 0.943 | 0.976 | ≈ | 0.50% |
| PCaeC34:0 | 1.227 (0.357) | 1.305 (0.436) | 0.474 | 0.939 | ↑ | 6.20% |
| PCaeC34:1 | 7.393 (1.970) | 7.641 (2.119) | 0.897 | 0.962 | ↑ | 3.30% |
| PCaeC34:2 | 7.223 (2.352) | 6.841 (1.746) | 0.499 | 0.939 | ↓ | 5.43% |
| PCaeC36:1 | 9.473 (2.362) | 10.03 (2.77) | 0.432 | 0.939 | ↑ | 5.71% |
| PCaeC36:2 | 9.617 (2.613) | 9.844 (2.548) | 0.748 | 0.939 | ↑ | 2.33% |
| PCaeC36:3 | 4.856 (1.356) | 4.501 (0.970) | 0.272 | 0.939 | ↓ | 7.58% |
| PCaeC36:5 | 8.063 (2.764) | 7.844 (2.152) | 0.745 | 0.939 | ↓ | 2.76% |
| PCaeC38:4 | 8.901 (2.105) | 8.900 (1.965) | 0.998 | 0.998 | ≈ | 0.01% |
| PCaeC38:5 | 11.32 (3.18) | 10.95 (2.54) | 0.917 | 0.966 | ↓ | 3.39% |
| SMC16:0 | 57.12 (15.25) | 57.20 (11.88) | 0.983 | 0.998 | ≈ | 0.14% |
| SMC18:0 | 15.50 (3.71) | 14.73 (3.45) | 0.435 | 0.939 | ↓ | 5.07% |
| H1 | 5568.9 (686.2) | 5333.2 (941.4) | 0.076 | 0.897 | ↓ | 4.32% |

Significant p-values are shown in bold. *p < 0.05 control vs delirium. q-values are from Benjamini–Hochberg. SD: standard deviation; Ala: alanine; Arg: arginine; Asn: asparagine; Asp: aspartate; Cit: citrulline ; Gln: glutamine; Glu: glutamate; Gly: glycine; His: histidine; Ile: isoleucine; Leu: leucine; Lys: lysine; Met: methionine; Orn: ornithine; Phe: phenylalanine; Pro: proline; Ser: serine; Thr: threonine; Trp: tryptophan; Tyr: tyrosine; Val: valine; ADMA: asymmetric dimethylarginine; SDMA: symmetric dimethylarginine; H1: hexose.
