# Supplementary material for: Blood–Brain Barrier Permeability in Cases of Post-operative Delirium Is Associated with Central Nervous System Phosphatidylcholine Imbalances
Source: Mol Neurobiol. 2026 Apr 21;63(1):575. doi: 10.1007/s12035-026-05847-3 (PMC13099853; doi:10.1007/s12035-026-05847-3)
Supplement: Supplementary file 4 — (DOCX 23.2 KB) [file 12035_2026_5847_MOESM4_ESM.docx]

**Supplementary Table 4. Univariate analysis between control and delirium in Qmetab**

| Metabolites | Control | Delirium | p-value | | q-value | ↑/↓ | %  difference |
| --- | --- | --- | --- | --- | --- | --- | --- |
|  | Mean (SD) | Mean (SD) |  |  |  |  |  |
| C3 | 0.1338 (0.0716) | 0.1309 (0.0891) | 0.775 | 0.897 | | ↓ | 2.19% |
| Ala | 0.1194 (0.0339) | 0.1300 (0.0473) | 0.396 | 0.708 | | ↑ | 8.47% |
| Arg | 0.2770 (0.0434) | 0.2987(0.0679) | 0.307 | 0.647 | | ↑ | 7.52% |
| Asn | 0.1720 (0.0413) | 0.1774 (0.0481) | 0.829 | 0.936 | | ↑ | 3.12% |
| Asp | 0.3448 (0.1014) | 0.3535 (0.0832) | 0.736 | 0.897 | | ↑ | 2.48% |
| Cit | 0.0820 (0.0185) | 0.0835 (0.0221) | 0.769 | 0.897 | | ↑ | 1.84% |
| Gln | 0.7696 (0.0747) | 0.8428 (0.1473) | **0.020*** | 0.325 | | ↑ | 9.09% |
| Glu | 0.0316 (0.0116) | 0.0348 (0.0171) | 0.904 | 0.936 | | ↑ | 9.66% |
| Gly | 0.0467 (0.0114) | 0.0488 (0.0239) | 0.580 | 0.877 | | ↑ | 4.53% |
| His | 0.1809 (0.0248) | 0.2013 (0.0322) | **0.014*** | 0.325 | | ↑ | 10.68% |
| Ile | 0.0942 (0.0219) | 0.1059 (0.0270) | 0.077 | 0.434 | | ↑ | 11.67% |
| Leu | 0.1096 (0.0324) | 0.1237 (0.0418) | 0.132 | 0.469 | | ↑ | 12.09% |
| Lys | 0.1771 (0.0295) | 0.1824 (0.0346) | 0.548 | 0.874 | | ↑ | 2.96% |
| Met | 0.1788 (0.0288) | 0.2003 (0.0447) | **0.042*** | 0.354 | | ↑ | 11.36% |
| Orn | 0.0904 (0.0211) | 0.0900 (0.0181) | 0.904 | 0.936 | | ≈ | 0.48% |
| Phe | 0.1781 (0.0331) | 0.1974 (0.0354) | **0.032*** | 0.325 | | ↑ | 10.25% |
| Pro | 0.0041 (0.0016) | 0.0047 (0.0028) | 0.640 | 0.878 | | ↑ | 13.22% |
| Ser | 0.2904 (0.0443) | 0.3125 (0.0597) | 0.151 | 0.469 | | ↑ | 7.30% |
| Thr | 0.2974 (0.0578) | 0.3111 (0.0561) | 0.382 | 0.704 | | ↑ | 4.49% |
| Trp | 0.0563 (0.0149) | 0.0581 (0.0122) | 0.368 | 0.700 | | ↑ | 3.19% |
| Tyr | 0.1688 (0.0297) | 0.1865 (0.0295) | **0.033*** | 0.325 | | ↑ | 9.95% |
| Val | 0.0886 (0.0176) | 0.1024 (0.0227) | **0.015*** | 0.325 | | ↑ | 14.49% |
| ADMA | 0.1686 (0.1235) | 0.2059 (0.1026) | 0.062 | 0.420 | | ↑ | 19.90% |
| Creatinine | 1.5697 (0.6778) | 1.7689 (0.9341) | 0.703 | 0.897 | | ↑ | 11.93% |
| Kynurenine | 0.0681 (0.0172) | 0.0684 (0.0171) | 0.964 | 0.964 | | ≈ | 0.31% |
| Met-SO | 0.0757 (0.0278) | 0.0792 (0.0334) | 0.710 | 0.897 | | ↑ | 4.53% |
| Putrescine | 1.2279 (0.5771) | 1.4867 (0.6093) | 0.064 | 0.420 | | ↑ | 19.07% |
| Spermidine | 0.5079 (0.1545) | 0.5777 (0.1504) | 0.141 | 0.469 | | ↑ | 12.86% |
| Spermine | 0.4061 (0.0611) | 0.3949 (0.0489) | 0.250 | 0.590 | | ↓ | 2.81% |
| t4-OH-Pro | 0.0646 (0.0192) | 0.0679 (0.0213) | 0.640 | 0.878 | | ↑ | 5.03% |
| Taurine | 0.1317 (0.0338) | 0.1491 (0.0376) | 0.081 | 0.434 | | ↑ | 12.36% |
| SDMA | 0.3863 (0.2529) | 0.5827 (0.7965) | 0.332 | 0.675 | | ↑ | 40.54% |
| PCaaC32:0 | 0.0338 (0.0138) | 0.0309 (0.0086) | 0.467 | 0.810 | | ↓ | 9.03% |
| PCaaC32:1 | 0.0091 (0.0044) | 0.0102 (0.0037) | 0.188 | 0.498 | | ↑ | 12.30% |
| PCaaC34:1 | 0.0091 (0.0030) | 0.0096 (0.0030) | 0.490 | 0.826 | | ↑ | 6.07% |
| PCaaC34:2 | 0.0006 (0.0002) | 0.0007 (0.0005) | 0.171 | 0.480 | | ↑ | 22.88% |
| PCaaC36:1 | 0.0063 (0.0028) | 0.0063 (0.0022) | 0.580 | 0.877 | | ≈ | 0.56% |
| PCaaC36:2 | 0.0011 (0.0004) | 0.0013 (0.0007) | 0.151 | 0.469 | | ↑ | 18.25% |
| PCaaC36:3 | 0.0006 (0.0003) | 0.0009 (0.0006) | 0.139 | 0.469 | | ↑ | 29.65% |
| PCaaC36:4 | 0.0012 (0.0005) | 0.0014 (0.0006) | 0.166 | 0.480 | | ↑ | 16.44% |
| PCaaC38:3 | 0.0012 (0.0006) | 0.0015 (0.0007) | 0.111 | 0.469 | | ↑ | 22.49% |
| PCaaC38:4 | 0.0021 (0.0009) | 0.0024 (0.0010) | 0.194 | 0.498 | | ↑ | 13.89% |
| PCaaC38:5 | 0.0011 (0.0004) | 0.0013 (0.0007) | 0.307 | 0.647 | | ↑ | 17.34% |
| PCaaC38:6 | 0.0008 (0.0004) | 0.0011 (0.0006) | 0.090 | 0.443 | | ↑ | 32.24% |
| PCaaC40:4 | 0.0048 (0.0033) | 0.0057 (0.0036) | 0.346 | 0.680 | | ↑ | 17.08% |
| PCaaC40:5 | 0.0017 (0.0012) | 0.0018 (0.0014) | 0.755 | 0.897 | | ↑ | 6.37% |
| PCaeC32:1 | 0.0083 (0.0049) | 0.0092 (0.0074) | 0.607 | 0.878 | | ↑ | 10.21% |
| PCaeC34:0 | 0.0119 (0.0077) | 0.0115 (0.0092) | 0.533 | 0.874 | | ↓ | 3.84% |
| PCaeC34:1 | 0.0093 (0.0032) | 0.0094 (0.0038) | 0.945 | 0.961 | | ≈ | 0.71% |
| PCaeC34:2 | 0.0050 (0.0025) | 0.0067 (0.0030) | **0.030*** | 0.325 | | ↑ | 28.28% |
| PCaeC36:1 | 0.0040 (0.0020) | 0.0043 (0.0024) | 0.631 | 0.878 | | ↑ | 6.94% |
| PCaeC36:2 | 0.0020 (0.0011) | 0.0022 (0.0013) | 0.688 | 0.897 | | ↑ | 6.18% |
| PCaeC36:3 | 0.0020 (0.0015) | 0.0025 (0.0023) | 0.729 | 0.897 | | ↑ | 20.46% |
| PCaeC36:5 | 0.0021 (0.0013) | 0.0021 (0.0016) | 0.892 | 0.936 | | ↑ | 2.61% |
| PCaeC38:4 | 0.0017 (0.0010) | 0.0018 (0.0013) | 0.845 | 0.936 | | ↑ | 3.46% |
| PCaeC38:5 | 0.0015 (0.0009) | 0.0019 (0.0009) | 0.115 | 0.469 | | ↑ | 24.52% |
| SMC16:0 | 0.0030 (0.0010) | 0.0029 (0.0013) | 0.203 | 0.499 | | ↓ | 4.40% |
| SMC18:0 | 0.0112 (0.0046) | 0.0116 (0.0045) | 0.863 | 0.936 | | ↑ | 2.95% |
| H1 | 0.7130 (0.2481) | 0.7646 (0.2145) | 0.283 | 0.642 | | ↑ | 6.98% |

Significant p-values are shown in bold. *p < 0.05 control vs delirium. q-values are from Benjamini–Hochberg. SD: standard deviation; Ala: alanine; Arg: arginine; Asn: asparagine; Asp: aspartate; Cit: citrulline ; Gln: glutamine; Glu: glutamate; Gly: glycine; His: histidine; Ile: isoleucine; Leu: leucine; Lys: lysine; Met: methionine; Orn: ornithine; Phe: phenylalanine; Pro: proline; Ser: serine; Thr: threonine; Trp: tryptophan; Tyr: tyrosine; Val: valine; ADMA: asymmetric dimethylarginine; SDMA: symmetric dimethylarginine; H1: hexose.
