# Supplementary material for: Blood–Brain Barrier Permeability in Cases of Post-operative Delirium Is Associated with Central Nervous System Phosphatidylcholine Imbalances
Source: Mol Neurobiol. 2026 Apr 21;63(1):575. doi: 10.1007/s12035-026-05847-3 (PMC13099853; doi:10.1007/s12035-026-05847-3)
Supplement: Supplementary file 5 — (DOCX 26.6 KB) [file 12035_2026_5847_MOESM5_ESM.docx]

**Supplementary Table 5. Correlation of the Qmetab with Qalb ratio**

| Metabolite | All participants | | | Control | | | Delirium | | | | |
| --- | --- | --- | --- | --- | --- | --- | --- | --- | --- | --- | --- |
|  | r | p-value | q-value | r | p-value | q-value | r | p-value | q-value | | |
| C3 | -0.0119 | 0.9334 | 0.9735 | -0.0345 | 0.8670 | 0.9837 | 0.0407 | 0.8435 | | 0.8580 | |
| Ala | 0.4496 | **0.0008^***^** | **0.0076^**^** | 0.4468 | **0.0221^*^** | 0.2858 | 0.4564 | **0.0191^*^** | | 0.0810 |  |
| Arg | 0.1055 | 0.4567 | 0.5733 | -0.0927 | 0.6526 | 0.9027 | 0.2827 | 0.1617 | | 0.2890 |  |
| Asn | 0.3276 | **0.0177^*^** | 0.0583 | 0.3326 | 0.0968 | 0.6109 | 0.2827 | 0.1617 | | 0.2890 |  |
| Asp | 0.1016 | 0.4826 | 0.5829 | 0.0661 | 0.7590 | 0.9370 | 0.1303 | 0.5259 | | 0.6465 |  |
| Cit | 0.3624 | **0.0083^**^** | **0.0445^*^** | 0.5699 | **0.0024^**^** | 0.0699 | 0.1720 | 0.4009 | | 0.5256 |  |
| Gln | 0.0861 | 0.5439 | 0.6418 | 0.0783 | 0.7038 | 0.9027 | -0.0038 | 0.9855 | | 0.9855 |  |
| Glu | -0.1090 | 0.4417 | 0.5665 | -0.1439 | 0.4830 | 0.7896 | -0.0927 | 0.6526 | | 0.7001 |  |
| Gly | 0.3246 | **0.0189^*^** | 0.0583 | 0.4332 | **0.0271^*^** | 0.2858 | 0.2485 | 0.2208 | | 0.3429 |  |
| His | 0.1887 | 0.1803 | 0.2727 | -0.0010 | 0.9960 | 0.9960 | 0.3128 | 0.1197 | | 0.2665 |  |
| Ile | 0.3130 | **0.0239^*^** | 0.0612 | 0.3046 | 0.1303 | 0.6162 | 0.3094 | 0.1240 | | 0.2665 |  |
| Leu | 0.1933 | 0.1698 | 0.2707 | -0.0010 | 0.9960 | 0.9960 | 0.3039 | 0.1312 | | 0.2665 |  |
| Lys | 0.2121 | 0.1311 | 0.2344 | 0.0892 | 0.6647 | 0.9027 | 0.3819 | 0.0542 | | 0.1513 |  |
| Met | 0.2040 | 0.1469 | 0.2476 | 0.0790 | 0.7014 | 0.9027 | 0.2650 | 0.1908 | | 0.3298 |  |
| Orn | 0.0015 | 0.9919 | 0.9919 | -0.2609 | 0.1981 | 0.6226 | 0.1966 | 0.3358 | | 0.4607 |  |
| Phe | 0.3177 | **0.0217^*^** | 0.0583 | 0.0865 | 0.6744 | 0.9027 | 0.4462 | **0.0223^*^** | | 0.0879 |  |
| Pro | 0.2945 | **0.0341^*^** | 0.0804 | 0.2533 | 0.2118 | 0.6247 | 0.2920 | 0.1478 | | 0.2812 |  |
| Ser | 0.1476 | 0.2965 | 0.3887 | 0.0564 | 0.7843 | 0.9370 | 0.1118 | 0.5866 | | 0.6531 |  |
| Thr | 0.3404 | **0.0135^*^** | 0.0533 | 0.4496 | **0.0212^*^** | 0.2858 | 0.2000 | 0.3273 | | 0.4598 |  |
| Trp | 0.0992 | 0.4841 | 0.5829 | 0.0537 | 0.7945 | 0.9370 | 0.0797 | 0.6989 | | 0.7363 |  |
| Tyr | 0.3200 | **0.0208^*^** | 0.0583 | 0.2274 | 0.2640 | 0.6463 | 0.3832 | 0.0533 | | 0.1513 |  |
| Val | 0.3413 | **0.0133^*^** | 0.0533 | 0.3108 | 0.1223 | 0.6162 | 0.3491 | 0.0805 | | 0.1929 |  |
| ADMA | 0.0067 | 0.9621 | 0.9787 | 0.1929 | 0.3452 | 0.6789 | -0.2622 | 0.1956 | | 0.3298 |  |
| Creatinine | 0.0254 | 0.8579 | 0.9373 | 0.1002 | 0.6263 | 0.9027 | -0.0571 | 0.7818 | | 0.8092 |  |
| Kynurenine | -0.0489 | 0.7305 | 0.8289 | 0.1631 | 0.4260 | 0.7617 | -0.2526 | 0.2131 | | 0.3397 |  |
| Met-SO | 0.1843 | 0.2256 | 0.3169 | 0.2381 | 0.2739 | 0.6463 | 0.1112 | 0.6221 | | 0.6797 |  |
| Putrescine | 0.2538 | 0.0694 | 0.1365 | 0.2684 | 0.1850 | 0.6226 | 0.1200 | 0.5593 | | 0.6470 |  |
| Spermidine | 0.0776 | 0.5847 | 0.6764 | -0.0612 | 0.7665 | 0.9370 | 0.1200 | 0.5593 | | 0.6470 |  |
| Seprmine | 0.0106 | 0.9405 | 0.9735 | -0.1357 | 0.5085 | 0.7896 | 0.2093 | 0.3049 | | 0.4497 |  |
| t4-OH-Pro | 0.2979 | **0.0320^*^** | 0.0786 | 0.1371 | 0.5042 | 0.7896 | 0.3921 | **0.0476^*^** | | 0.1513 |  |
| Taurine | 0.1759 | 0.2123 | 0.3055 | 0.0817 | 0.6915 | 0.9027 | 0.1125 | 0.5843 | | 0.6531 |  |
| SDMA | 0.1530 | 0.2789 | 0.3739 | 0.0297 | 0.8853 | 0.9855 | 0.2267 | 0.2655 | | 0.4016 |  |
| PCaaC32:0 | 0.1894 | 0.1788 | 0.2727 | -0.0120 | 0.9537 | 0.9960 | 0.4598 | **0.0181^*^** | | 0.0810 |  |
| PCaaC32:1 | 0.2082 | 0.1386 | 0.2405 | -0.0496 | 0.8100 | 0.9370 | 0.3928 | **0.0471^*^** | | 0.1513 |  |
| PCaaC34:1 | 0.2601 | 0.0626 | 0.1273 | -0.0229 | 0.9116 | 0.9960 | 0.5480 | **0.0038^**^** | | **0.0246^*^** |  |
| PCaaC34:2 | 0.5621 | **1.45E-05^***^** | **0.0003^***^** | 0.4277 | 0.0293 | 0.2858 | 0.6568 | **0.0003^***^** | | **0.0053^**^** |  |
| PCaaC36:1 | 0.1829 | 0.1943 | 0.2866 | 0.0147 | 0.9432 | 0.9960 | 0.2581 | 0.2030 | | 0.3326 |  |
| PCaaC36:2 | 0.5137 | **9.83E-05^***^** | **0.0015^**^** | 0.2171 | 0.2867 | 0.6499 | 0.7621 | **6.08E-06^***^** | | **0.0004^***^** |  |
| PCaaC36:3 | 0.6331 | **4.74E-07^***^** | **2.80E-05^***^** | 0.6294 | **0.0006^***^** | **0.0336^*^** | 0.6198 | **0.0007^***^** | | **0.0108^*^** |  |
| PCaaC36:4 | 0.4613 | **0.0006^***^** | **0.0068^**^** | 0.2732 | 0.1770 | 0.6226 | 0.5685 | **2.44E-03^***^** | | **0.0206^**^** |  |
| PCaaC38:3 | 0.5682 | **1.11E-05^***^** | **0.0003^***^** | 0.3265 | 0.1036 | 0.6109 | 0.7251 | **2.78E-05^***^** | | **0.0008^***^** |  |
| PCaaC38:4 | 0.4125 | **0.0024^**^** | **0.0141^*^** | 0.1460 | 0.4767 | 0.7896 | 0.5591 | **0.0030^**^** | | **0.0221^*^** |  |
| PCaaC38:5 | 0.4466 | **0.0009^***^** | **0.0076^**^** | 0.2465 | 0.2248 | 0.6315 | 0.5829 | **0.0018^**^** | | **0.0175^*^** |  |
| PCaaC38:6 | 0.4122 | **0.0024^**^** | **0.0141^*^** | 0.4117 | **0.0367^*^** | 0.2858 | 0.3812 | 0.0547 | | 0.1513 |  |
| PCaaC40:4 | 0.2660 | 0.0566 | 0.1193 | -0.0024 | 0.9907 | 0.9960 | 0.4560 | **0.0192^*^** | | 0.0810 |  |
| PCaaC40:5 | 0.2823 | **0.0426^*^** | 0.0931 | 0.4076 | **0.0388^*^** | 0.2858 | 0.1251 | 0.5424 | | 0.6470 |  |
| PCaeC32:1 | 0.4376 | **0.0012^*^** | **0.0087^**^** | 0.2617 | 0.1965 | 0.6226 | 0.5857 | **0.0017^**^** | | **0.0175^*^** |  |
| PCaeC34:0 | 0.0338 | 0.8121 | 0.9040 | -0.1833 | 0.3701 | 0.7043 | 0.3007 | 0.1355 | | 0.2665 |  |
| PCaeC34:1 | 0.3498 | **0.0110^*^** | 0.0533 | 0.3005 | 0.1358 | 0.6162 | 0.3525 | 0.0774 | | 0.1929 |  |
| PCaeC34:2 | 0.3419 | **0.0131^*^** | 0.0533 | 0.1782 | 0.3839 | 0.7078 | 0.4120 | **0.0365^*^** | | 0.1346 |  |
| PCaeC36:1 | 0.2495 | 0.0745 | 0.1417 | 0.2125 | 0.2974 | 0.6499 | 0.2048 | 0.3155 | | 0.4540 |  |
| PCaeC36:2 | 0.3359 | **0.0149^*^** | 0.0550 | 0.1583 | 0.4399 | 0.7633 | 0.5085 | **0.0080^**^** | | **0.0471^*^** |  |
| PCaeC36:3 | 0.3223 | **0.0198^*^** | 0.0583 | 0.2077 | 0.3087 | 0.6505 | 0.3787 | 0.0564 | | 0.1513 |  |
| PCaeC36:5 | 0.3232 | **0.0194^*^** | 0.0583 | 0.2653 | 0.1902 | 0.6226 | 0.3477 | 0.0817 | | 0.1929 |  |
| PCaeC38:4 | 0.1569 | 0.2666 | 0.3659 | 0.2291 | 0.2602 | 0.6463 | 0.1728 | 0.3987 | | 0.5256 |  |
| PCaeC38:5 | 0.2888 | **0.0378^*^** | 0.0859 | 0.1077 | 0.6004 | 0.9027 | 0.4794 | **0.0132^*^** | | 0.0709 |  |
| SMC16:0 | 0.2438 | 0.0816 | 0.1505 | 0.2410 | 0.2356 | 0.6318 | 0.3067 | 0.1276 | | 0.2665 |  |
| SMC18:0 | 0.0155 | 0.9129 | 0.9735 | -0.1938 | 0.3427 | 0.6789 | 0.1364 | 0.5064 | | 0.6357 |  |
| H1 | -0.1953 | 0.1653 | 0.2707 | -0.2595 | 0.2005 | 0.6226 | -0.1562 | 0.4460 | | 0.5720 |  |

R-values are from either Pearson or Spearman correlation and represent the correlation coefficient r between metabolite ratio and Qalb ratio. Significant p-values are shown in bold. *p < 0.05, **p < 0.01, *** p < 0.001 metabolite ratio vs Qalb ratio. q-values are from Benjamini–Hochberg. Significant q-values are shown in bold. *q < 0.05, **q < 0.01, *** q < 0.001 metabolite ratio vs Qalb ratio. Ala: alanine; Arg: arginine; Asn: asparagine; Asp: aspartate; Cit: citrulline; Gln: glutamine; Glu: glutamate; Gly: glycine; His: histidine; Ile: isoleucine; Leu: leucine; Lys: lysine; Met: methionine; Orn: ornithine; Phe: phenylalanine; Pro: proline; Ser: serine; Thr: threonine; Trp: tryptophan; Tyr: tyrosine; Val: valine; ADMA: asymmetric dimethylarginine; SDMA: symmetric dimethylarginine; H1: hexose.
