# Supplementary material for: Blood–Brain Barrier Permeability in Cases of Post-operative Delirium Is Associated with Central Nervous System Phosphatidylcholine Imbalances
Source: Mol Neurobiol. 2026 Apr 21;63(1):575. doi: 10.1007/s12035-026-05847-3 (PMC13099853; doi:10.1007/s12035-026-05847-3)
Supplement: Supplementary file 6 — (DOCX 16.4 KB) [file 12035_2026_5847_MOESM6_ESM.docx]

**Supplementary Table 6.** **The univariate analysis of lipid concentrations in CSF between e4 yes and no.**

| Metabolites | E4 (Yes) (*n*=36)  Mean (SD) | E4 (No) (*n*=17)  Mean (SD) | p-value |
| --- | --- | --- | --- |
| PCaaC32:0 | 0.3136 (0.1012) | 0.3241 (0.0583) | 0.706 |
| PCaaC32:1 | 0.1442 (0.0513) | 0.1388 (0.0265) | 0.759 |
| PCaaC34:1 | 1.9789 (0.5906) | 1.9447 (0.3599) | 0.723 |
| PCaaC34:2 | 0.2025 (0.1307) | 0.1814 (0.0601) | 0.306 |
| PCaaC36:1 | 0.2306 (0.0746) | 0.2196 (0.0463) | 0.737 |
| PCaaC36:2 | 0.2054 (0.1094) | 0.1872 (0.0606) | 0.153 |
| PCaaC36:3 | 0.0828 (0.0564) | 0.0680 (0.0295) | 0.918 |
| PCaaC36:4 | 0.2117 (0.1397) | 0.1907 (0.0607) | 1.000 |
| PCaaC38:3 | 0.0547 (0.0285) | 0.0501 (0.0225) | 0.220 |
| PCaaC38:4 | 0.1926 (0.1026) | 0.1761 (0.0549) | 0.540 |
| PCaaC38:5 | 0.0554 (0.0305) | 0.0461 (0.0104) | 0.183 |
| PCaaC38:6 | 0.0605 (0.0569) | 0.0515 (0.0211) | 0.609 |
| PCaaC40:4 | 0.0122 (0.0102) | 0.0154 (0.0078) | 0.758 |
| PCaaC40:5 | 0.0148 (0.0113) | 0.0175 (0.0125) | 0.357 |
| PCaeC32:1 | 0.0212 (0.0121) | 0.0149 (0.0093) | 0.101 |
| PCaeC34:0 | 0.0139 (0.0095) | 0.0132 (0.0079) | 0.098 |
| PCaeC34:1 | 0.0673 (0.0234) | 0.0651 (0.0154) | 0.530 |
| PCaeC34:2 | 0.0428 (0.0253) | 0.0319 (0.0109) | 0.608 |
| PCaeC36:1 | 0.0425 (0.0257) | 0.0346 (0.0204) | 0.759 |
| PCaeC36:2 | 0.0200 (0.0116) | 0.0192 (0.0114) | 0.838 |
| PCaeC36:3 | 0.0124 (0.0118) | 0.0073 (0.0047) | 0.411 |
| PC ae C36:5 | 0.0168 (0.0113) | 0.0136 (0.0092) | 0.681 |
| PCaeC38:4 | 0.0169 (0.0120) | 0.0137 (0.0109) | 0.837 |
| PCaeC38:5 | 0.0201 (0.0121) | 0.0148 (0.0085) | 0.609 |
| SMC16:0 | 0.1618 (0.0724) | 0.1676 (0.0480) | 0.153 |
| SMC18:0 | 0.1664 (0.0587) | 0.1614 (0.0485) | 0.570 |
